# The relationship between baseline nutritional status with subsequent parenteral nutrition and clinical outcomes in cancer patients undergoing cytoreductive surgery: a retrospective study

**DOI:** 10.3389/fnut.2024.1364959

**Published:** 2024-05-03

**Authors:** Esraa AlTawil, Nora A. Kalagi, Sohailah Alzahrani, Faisal Alobeed, Sulaiman Alshammari, Thamer Bin Traiki

**Affiliations:** ^1^Clinical Pharmacy Services, Pharmacy Department, King Saud University Medical City, Riyadh, Saudi Arabia; ^2^Department of Clinical Pharmacy, College of Pharmacy, King Saud University, Riyadh, Saudi Arabia; ^3^Department of Clinical Pharmacy, Prince Sultan Military Medical City, Riyadh, Saudi Arabia; ^4^College of Medicine, King Saud Bin Abdulaziz University for Health Sciences, Riyadh, Saudi Arabia; ^5^Colorectal Research Chair, Department of Surgery, College of Medicine, King Saud University, Riyadh, Saudi Arabia

**Keywords:** nutritional status, cancer, parenteral nutrition, cytoreductive surgery, hyperthermic

## Abstract

**Introduction:**

Hyperthermic Intraperitoneal Chemotherapy (HIPEC) with Cytoreductive Surgery (CRS) is the preferred treatment for peritoneal malignancies. This highly complex operation is associated with a high incidence of complications, particularly due to malnutrition. This study aimed to investigate the potential association between preoperative nutritional status and postoperative clinical outcomes in adult cancer patients who underwent CRS/HIPEC for peritoneal malignancy.

**Methods:**

A retrospective study with 140 adult cancer patients, on parenteral nutrition (PN) (*n* = 40) and not on PN (*n* = 100) who underwent CRS with or without HIPEC, was conducted.

**Results:**

Patients who received PN had significantly longer post-operative, hospital, and ICU LOS than those who did not (*p* = 0.001). ICU admission was significantly higher in the non-PN receiving group compared to the PN receiving group. When compared to the PN group, the majority of patients not receiving PN were at low risk of malnutrition (91% vs. 75%, *p* = 0.020), whereas 17.5% of PN patients were at risk of malnutrition during hospitalization. Multiple regression analyses revealed a strong positive relationship between patients with increased risk of malnutrition and ICU LOS (*p* = 0.047).

**Discussion:**

Routine preoperative nutrition assessment is essential to identify patients who are at higher nutritional risk, and nutrition support should be provided preoperatively.

## 1 Introduction

Hyperthermic Intraperitoneal Chemotherapy (HIPEC) with Cytoreductive Surgery (CRS) is the preferred treatment for peritoneal mesothelioma, pseudomyxoma peritonei, peritoneal carcinomatosis, and other peritoneal malignancies (1). The CRS aims to reduce the residual tumor volume by the aggressive resection of visceral and peritoneal components, followed by HIPEC administration to minimize the incidence of postoperative adverse events. This complex medical operation is associated with a high incidence of significant gastrointestinal complications. Patients with abdominopelvic malignancy, who undergo CRS/HIPEC, experience high risk of mortality, prolonged hospital length of stay (LOS), and morbidity, particularly due to malnutrition (2). Multiple evidence have also revealed a 0.9–5.8% mortality rate and a 12–60% morbidity rate following the implementation of the CRS/HIPEC procedure (3). Most importantly, chemotherapy can further add to the incidence of systemic toxicity and postoperative complications. Evidence have demonstrated that these outcomes have a detrimental impact on the overall health-related quality of life and nutritional status of the treated patients (4).

Peritoneal carcinomas have been reported to be associated with 60–80% incidence of malnutrition. This emphasizes the significance of providing appropriate nutrition support to minimize the risk of clinical complications (5, 6). Nonetheless, evidence in the current literature concerning the clinically significant relationship between postoperative outcomes and the nutrition status of patients treated with CRS/HIPEC is scarce (7). Recent retrospective study has demonstrated enteral nutrition to be a protective factor for LOS in patients with pseudomyxoma peritonei treated with CRS/HIPEC (8). The conclusion from another real-world study has also indicated the possible association of the postoperative parenteral nutrition (PN) requirement with the operative and nutritional factors in the setting of CRS (9). Furthermore, findings from other studies emphasized poor nutritional status as a preoperative factor, which deteriorates the postoperative clinical outcomes after CRS/HIPEC administration (10). A prospective observational study has additionally reported a clinically significant relationship between postoperative outcomes, such as LOS and infections, and preoperative malnutrition in patients with peritoneal malignancies (2). This great amount of evidence claimed the importance of optimizing perioperative nutritional support to reduce the incidence of postoperative complications and improve postoperative recovery in patients undergoing CRS/HIPEC (11). This could be potentially established by statistically comparing preoperative and postoperative nutritional parameters. To the best of our knowledge, no real-world studies have yet established a statistically meaningful association between PN and postoperative adverse events, comparing PN-related versus non-PN-related complications and their correlation with patients’ preoperative (baseline) nutritional status in patients undergoing CRS/HIPEC. This virgin area of research could significantly enhance nutrition-based risk stratification, prognosis, and recovery patterns in patients requiring CRS/HIPEC. Furthermore, the assessment of the relationship between postoperative PN duration/optimal timing and patient’s baseline nutrition levels is paramount to determining the high-risk patients (i.e., those with an increased predisposition for postoperative/hospital/intensive care unit (ICU) LOS and mortality). Improved clinical decision-making through nutritional screening could also effectively minimize postoperative outcomes in CRS/HIPEC patients, including the occurrence of serious/life-threatening complications. This single-center study was aimed to investigate the possible association between the preoperative nutritional status and postoperative clinical outcomes, as well as the incidence of PN-related and non-PN-related postoperative complications in adult cancer patients who underwent CRS/HIPEC for peritoneal malignancy.

## 2 Materials and methods

### 2.1 Study design and population

This single-center retrospective study has enrolled adult cancer patients (age > 18 years) who underwent CRS with or without HIPEC monotherapy, between January 2017 and December 2022 at King Saud University Medical City (KSUMC), Kingdom of Saudi Arabia. All included study participants had a clinician confirmed diagnosis of colorectal, gynecologic, appendiceal, and/or peritoneal malignancies. Study participants were divided into two groups: patients who received PN after procedure (i.e., PN group *n* = 40) and patients who did not receive PN after procedure (i.e., non-PN group *n* = 100). Patients started PN after being screened for their nutritional status using validated tools such as the Nutrition Risk Screening (NRS)-2002. Patients deemed at high risk of malnutrition based on their NRS scores were considered candidates for PN therapy to optimize their nutritional status during the perioperative period. Moreover, clinician discretion played a role in the decision-making process, with healthcare providers considering various clinical factors and patient-specific variables including severity and extent of disease, surgical complexity and duration, pre-existing nutritional deficiencies and comorbidities, anticipated postoperative recovery, and potential risks associated with inadequate oral intake. Approval of the study protocol was obtained from KSUMC Institutional Review Board (E-23-7718) before the commencement of the study, and all procedures were performed in accordance with the Declaration of Helsinki.

### 2.2 Clinical data and assessment tools

After screening, data on patients’ demographics, baseline NRS, cancer site, type of surgery, postoperative and hospital LOS, ICU admission, oral intake, and enteral feeding were collected for all study participants. The primary outcome of interest was the association between baseline nutritional status and clinical outcomes in CRS/HIPEC patients who either received or did not receive PN. The secondary outcomes were postoperative PN-related complications, non-PN-related complications, and ICU admissions.

PN-related complications were defined as the composite of one or more of the following complications: hyperglycemia [fasting blood glucose>180 mg/dL (10 mmol/L)]; electrolyte imbalances, including Hyponatremia (Sodium <135 mmol/L), Hypernatremia (Sodium >145 mmol/L), Hypomagnesemia (Magnesium <0.7 mmol/L), Hypermagnesemia (Magnesium >1.10 mmol/L), Hypokalemia (Potassium <3.5 mmol/L), Hyperkalemia (Potassium >5.5 mmol/L), Hypophosphatemia (Phosphorus <0.8 mmol/L), Hyperphosphatemia (Phosphorus >1.45 mmol/L), Hypocalcemia (Calcium <2.2 mmol/L), and Hypercalcemia (Calcium >2.7 mmol/L); hypertriglyceridemia (Triglycerides >150 mg/dL); hepatic steatosis (diagnosed as elevation in biochemical markers: aspartate transaminase (AST), alanine transaminase (ALT) >2x UNL or imaging Studies); or cholestasis [defined as increasing in biochemical markers, such as Total bilirubin (>2 mg/dL), Alkaline phosphatase (>1.5 x ULN), and gamma-glutamyl transpeptidase (GGT) (>3 x ULN) in patients receiving PN, and not due to other liver diseases or biliary obstruction].

Non-PN-related complications have also been assessed, which were defined as the composite of one or more of the following complications: catheter-related [catheter related bloodstream infection (CRBSI), cerebral venous thrombosis (CVT), pulmonary embolism (PE), or occlusion], infectious [wound related infection, bacteremia, or urinary tract infection (UTI)], hematological (leukopenia, leukocytosis, thrombocytopenia, or thrombocytosis) gastrointestinal (intra-peritoneal complications or chyle leaks), renal (AKI or azotemia), or death.

### 2.3 Nutritional status

The Nutrition Risk Screening (NRS)-2002, a simple and well-validated tool had been used to assess patients’ nutritional status (12). It represented the sum of the nutritional, disease severity, and age adjustment scores. Scoring is categorized as Low-risk (score = 0–3: indicating weekly rescreening requirement), at-risk (score = 4, indicating the need to initiate nutritional care plan) (food, oral supplements, tube feeding, and/or parenteral nutrition as appropriate), and high-risk (score = 5–7, indicating the need to initiate early intervention nutritional care plan).

### 2.4 Statistical analysis

The patients’ characteristics were summarized according to PN status and measured using descriptive statistics. The continuous variables were expressed as mean ± standard deviation or median [interquartile range (IQR)] depending on the data distribution. While categorical variables were expressed as numbers (percentages). The student’s *t*-test was used to compare age, body mass index (BMI), and albumin level, whereas Mann–Whitney U-test was used to compare postoperative, hospital, and ICU LOS. Linear and logistic regression analyses were performed to measure the association between NRS and clinical outcomes in both PN groups. All data were analyzed using Statistical Package for Social Studies (SPSS v. 29; IBM Corp., New York, NY, United States).

## 3 Results

### 3.1 Baseline characteristics

A total of 140 patients who underwent CRS with or without HIPEC monotherapy or their combinations and met the study inclusion criteria were admitted in the study. Participants in the study were divided into two groups: PN group (*n* = 40) and non-PN group (*n* = 100). Demographic variables including age, gender, and BMI did not differ significantly between the two groups. However, significant difference was found in albumin levels (*p* = 0.029) among both groups. Colorectal cancer cases were higher and statistically significant in the non-PN group than in the PN group (62% vs. 37.5%, *p* = 0.002), however, appendix cancer cases were higher and statistically significant in the PN group (*p* = 0.002). The type of surgery performed in neither group was significantly different (*p* = 0.712). All the baseline characteristics of the study participants are presented in Table 1.

**Table 1:** Baseline characteristics of the study participants receiving and not receiving PN.

	Total (*n* = 140)	PN (*n* = 40)	Non-PN (*n* = 100)	*p*-value^*^
Age (years)^a^	53.14 ± 13	52 ± 12	53 ± 13	0.413
Gender, *N* (%)				0.280
Male	60 (42.9)	20 (50)	40 (40)	
Female	80 (57.1)	20 (50)	60 (60)	
BMI (Kg/m^2^)^a^	27.75 ± 6.15	25.71 ± 6.50	28.43 ± 6.03	0.326
Albumin level (g/l)^a^	25.56 ± 6.32	22.46 ± 7.071	26.8 ± 5.88	0.029^*^
Cancer Site, *N* (%)				0.002^*^
Colorectal	77 (55)	15 (37.5)	62 (62)^*^	
Gynecologic	19 (13.9)	7 (17.5)	12 (12)	
Appendix	28 (20)	14 (35)^*^	14 (14)	
Peritoneal/ mesothelioma	2 (1.4)	2 (5)	0	
Others^b^	14 (10)	2 (5)	12 (12)^*^	
Type of Surgery, *N* (%)				0.712
CRS	20 (14.3)	6 (15)	14 (14)	
CRS + HIPEC	108 (77.1)	30 (75)	78 (78)	
HIPEC	1 (0.7)	1 (2.5)	0	
CRS + IORT	7 (5)	2 (5)	5 (5)	
CRS+ HIPEC + IORT	4 (2.9)	1 (2.5)	3 (3)	

PN, parenteral nutrition; BMI, body mass index; CRS, Cytoreductive surgery; HIPEC, hyperthermic intraperitoneal chemotherapy; IORT, Combined Intraoperative Radiotherapy; ICU, intensive care unit; LOS, length of study; N, number; NRS, Nutrition Risk Screening 2002. ^*^Statistically significant difference between the PN groups (*p* < 0.05).

a Data are presented as mean ± standard deviation. Student’s *t* test was used in the comparison.

b Others, include: (Omentum, small intestine, stomach, neuroendocrine, unknown).

### 3.2 Postoperative nutritional and surgical outcomes

The postoperative nutritional and surgical outcomes for both study groups are shown in Table 2. Patients who received PN had significantly longer post-operative, hospital, and ICU LOS (*p* = 0.001) than those who did not receive PN. However, the number of ICU admission was considerably greater in the non-PN than in the PN group. When compared to those on PN, the majority of patients in the non-PN group were at low risk of malnutrition according to the NRS criteria (91% vs. 75%, *p* = 0.020). On the other hand, 17.5% of PN patients were at risk of malnutrition during hospitalization, compared to 4% of non-PN patients.

**Table 2:** Postoperative nutritional and surgical outcomes among PN and non- PN groups.

	Total (*n* = 140)	PN (*n* = 40)	Non-PN (*n* = 100)	*p*-value^*^
Post-operative LOS (days)^a^	16.50 (10–30)	32 (21–48)	14 (9–21)	<0.001^*^
Hospital LOS (days)^a^	21 (14–38)	44 (28–70)	18 (13–28)	<0.001^*^
ICU LOS (days)^a^	2 (1–3)	4 (2–6)	1 (0–2)	<0.001^*^
ICU admission, *N* (%)	109 (77.9)	38 (95)	71 (71)	<0.001^*^
NRS, *N* (%)				0.020^*^
Low risk	121 (86.4)	30 (75)	91 (91)^*^	
At risk	11 (7.9)	7 (17.5)^*^	4 (4)	
High risk	8 (5.7)	3 (7.5)	5 (5)	
Oral intake, *N* (%)	58 (41.4)	10 (25)	48 (48)	0.014^*^
Enteral Feeding, *N* (%)	18 (12.9)	4 (10)	14 (14)	0.370

PN, parenteral nutrition; ICU, intensive care unit; LOS, length of study; N, number; NRS, Nutrition Risk Screening 2002. ^*^Statistically significant difference between the PN groups (*p* < 0.05).

a Data are presented as median (p25-p75). Mann–Whitney U test was used in the comparison.

Subgroup analysis of the PN group for the parenteral nutrition formula composition revealed that the median duration of the PN was 11 (1, 7–16) days, with nearly 50% of the patients reaching the PN goal (Table 3).

**Table 3:** Composition of parenteral nutrition formula among PN group.

	PN + (*N* = 40)
PN duration (days)^a^	11 (7–17)
Volume (mL/day)^a^	1920 (1807–2,395)
Dextrose (gm/day)^b^	256.10 ± 84.77
Protein (gm/day)^b^	84.75 ± 24.05
Fat (gm/day)^a^	36 (24–48)
Kcal/kg/day^b^	27.33 ± 5.48
Reached Target, *N* (%)	19 (47.5)

PN, parenteral nutrition; Kcal, kilocalories; N, number.

a Data are presented as median (p25–p75).

b Data are presented as mean ± standard deviation.

### 3.3 PN and non-PN related complications

Electrolyte imbalance, metabolic, and hepatic complications were the most reported PN-related complications (Table 4). Hypernatremia, hypokalemia, hypophosphatemia, hypomagnesemia, and hypocalcemia were evident in the PN group. In 35% of the patients, PN was associated with hyperglycemia and, to a lesser extent, hypertriglyceridemia (10%). The most prevalent hepatic complications in the PN group were cholestasis and steatosis, with nearly 50% of patients on PN developing cholestasis.

**Table 4:** PN-related complications among patients receiving PN (*n* = 40).

PN related complications			
	Normal	Hypo	Hyper
Electrolyte imbalance^a^, *N (%)*			
Sodium	25 (62.5)	6 (15)	9 (22.5)
Potassium	23 (57.5)	13 (32.5)	4 (10)
Phosphorous	21 (52.3)	13 (52.5)	6 (15)
Magnesium	23 (57.5)	11 (27.5)	6 (15)
Calcium	28 (70)	9 (22.5)	3 (7.5)
Metabolic complications, *N (%)*			
Hyperglycemia^b^		14 (35)	
Hypertriglyceridemia^c^		4 (10)	
Hepatic complications, *N (%)*			
Cholestasis		20 (50)	
Steatosis		12 (30)	

a Electrolyte Imbalances: Sodium (Na+): Normal (135–145) mmol/L, Hypo <135 mmol/L, Hyper >145 mmol/L; Magnesium (Mg): Normal (0.7–1.10)mmol/L, Hypo <0.7 mmol/L, Hyper >1.10 mmol/L; Potassium (K): Normal (3.5–5.5) mmol/L, Hypo <3.5 mmol/L, Hyper >5.5 mmol/L; Phosphorus (Ph): Normal (0.8–1.45)mmol/L, Hypo <0.8 mmol/L, Hyper >1.45 mmol/L; Calcium (Ca): Normal (2.2–2.7) mmol/L, Hypo <2.2 mmol/L, Hyper >2.7 mmol/L.

b Fasting Blood Glucose Level > 180 mg/dL (10 mmol/L).

c Triglyceride level > 150 to 499 mg/dL (1.7 to 5.6 mmol/L).

Non-PN related complications have also been observed in both PN and non-PN patients, as shown in Table 5. Complications include catheter related, infectious, hematological, gastrointestinal, and renal complications. In comparison to the non-PN group, PN was associated with more incidents of catheter-related bloodstream infection (*p* = 0.006), wound-related infection (*p* = 0.002), bacteremia (*p* = 0.014), leukopenia (*p* = 0.005), thrombocytopenia (*p* = 0.029), intra-peritoneal abscess (*p* = 0.009), acute kidney injury, and azotemia. Deaths were also recorded in both groups, with the PN group having more cases (*p* = 0.047) than the non-PN group.

**Table 5:** Non- PN related complications among both PN groups.

	PN (*n* = 40)	Non-PN (*n* = 100)	*p*-value^*^
Catheter related complication, *N* (%)			
CRBSI	8 (20)	5 (5)	0.006^*^
CVT	0	0	
PE	1 (2.5)	0	0.113
Occlusion	1 (2)	2 (2)	0.854
Infectious complications, *N* (%)			
Wound-related infection	21 (52.5)	25 (25)	0.002^*^
Bacteremia	14 (35)	16 (16)	0.014^*^
UTI	2 (5)	7 (7)	0.663
Hematological complications, *N* (%)			
WBC (cells/mm^3^)^a^	5,950 (3300–9,450)	9,000 (6100–12,500)	0.005^*^
Normal	17 (42.5)	56 (56)	0.012^*^
Leukopenia	14 (35)^*^	13 (13)	
Leukocytosis	9 (22.5)	31 (31)	
Platelets (cells/mm^3^)			0.029^*^
≥100,000	28 (70)	89 (89)^*^	
75,000-99,000	6 (15)^*^	4 (4)	
50,000-74,000	3 (7.5)	6 (6)	
25,000-49,000	2 (5)	1 (1)	
<25,000	1 (2.5)	0	
Bleeding	19 (47.5)	43 (43)	0.628
GI abnormality, *N* (%)			
Intra-peritoneal abscesses	15 (37.5)	17 (17)	0.009^*^
Chyle leak	1 (2.5)	2 (2)	0.854
Renal complications, *N* (%)			
AKI	11 (27.5)	13 (13)	0.038^*^
Azotemia	10 (25)	8 (8)	0.010^*^
Death, *N* (%)	6 (15)	5 (5)	0.047^*^

PN, parenteral nutrition; N, number; CRBSI, Catheter-related bloodstream infection; CVT, Cerebral Venous Thrombosis (CVT); PE, Pulmonary Embolism; UTI, urinary tract infection; WBC, white blood cells; AKI, acute kidney injury. ^*^Statistically significant difference between the PN groups (*p* < 0.05).

a WBC: Data are presented as median (p25–p75) and N (%). Normal 4,000-11,000, Leukopenia <4,000, Leukocytosis >11,000.

### 3.4 Nutritional status and patient’s clinical outcome

As presented in Table 6, the association between the patients’ NRS and clinical outcomes including postoperative, hospital, and ICU LOS and death have been assessed in multiple regression analyses. Unadjusted models demonstrated a non-significant association between nutritional risk and LOS in all settings except the ICU. This demonstrates a strong positive relationship between increased risk of malnutrition and ICU LOS (*p* = 0.047). A stratified analysis based on PN status revealed a non-significant correlation between the patients’ NRS and clinical outcomes in the PN group only. However, in the non-PN group, the risk of malnutrition was positively linked with the ICU LOS (*p* = 0.008) but not with other outcomes.

**Table 6:** Association between patients NRS and clinical outcomes in multiple regression analyses and among PN groups individually.

Outcomes		Post-operative LOS^a^			Hospital LOS^a^			ICU LOS^a^			Death^b^		
		Β	Pvalue	95% CI	Β	Pvalue	95% CI	B	Pvalue	95% CI	B	Pvalue	95% CI
NRS	Low risk	1(Ref)			1(Ref)			1(Ref)			1(Ref)		
	At risk	−0.023	0.787	−50.574, 38.392	0.154	0.071	−1.673, 40.285	0.052	0.538	−3.589, 6.845	1.244	0.843	0.143, 10.844
	High risk	−0.053	0.539	−67.644, 35.485	0.032	0.704	−19.638, 28.999	0.1619	0.047^*^	0.069, 12.165	1.778	0.605	0.196, 16.086
PN group													
NRS	Low risk	1(Ref)			1(Ref)			1(Ref)			1(Ref)		
	At risk	−0.145	0.381	−115.656, 45.256	0.019	0.908	−42.422, 47.565	−0.075	0.639	−14.897, 9.259	1.083	0.947	0.102, 11.523
	High risk	−0.084	0.609	−145.599, 86.532	0.002	0.992	−64.574, 65.241	0.279	0.087	−2.290, 32.557	3.250	0.378	0.236, 44.691
Non-PN group													
NRS	Low risk	1(Ref)			1(Ref)			1(Ref)					
	At risk	0.009	0.931	−56.660, 61.864	0.171	0.092	−2.323, 30.460	0.263	0.008^*^	0.781, 5.186			
	High risk	−0.061	0.551	−69.332, 37.235	0.010	0.923	−14.019, 15.456	−0.051	0.606	−2.497, 1.464			

a Beta coefficient computed using linear regression analysis.

b Beta coefficient representing the adjusted odd ratio (AOR) computed using logistic regression analysis.

^*^Statistically significant association (*p* < 0.05).

## 4 Discussion

This single-center retrospective study investigated the potential association between the preoperative nutritional status and postoperative clinical outcomes, as well as the incidence of PN and non-PN-related postoperative complications in adult cancer patients who underwent CRS/HIPEC for peritoneal malignancy. Patients who received PN had significantly longer post-operative, hospital, and ICU LOS compared to those who did not receive PN. Number of ICU admissions in the non-PN group was significantly higher than in the PN group. During hospitalization, the majority of the patients who did not receive PN were at low risk of malnutrition, whereas the majority of patients on PN were at risk of malnutrition. The association between the patients’ nutritional status and clinical outcomes showed a strong positive relationship between an increased risk of malnutrition and ICU LOS. Furthermore, the risk of malnutrition was positively linked with the ICU LOS in the non-PN group.

Preoperative nutrition status can have a significant impact on predicting postoperative outcomes (7) and complications in cancer patients undergoing CRS/HIPEC (10). The findings from several studies have suggested the significant association between clinical outcomes and PN use (2, 13, 14). Furthermore, a recent study by Khan et al. has suggested the importance of focusing on nutrition and improving its quality to limit the complications associated with the CRS/HIPEC (9). PN have also been found to influence the outcomes in terms of length of stay and survival, especially since patients after the surgeries suffer from an intolerance of oral intake within the first week. Therefore, optimizing and receiving the most adequate perioperative nutrition is required to enhance clinical outcomes. According to the systematic review of Gearing et al., Subjective Global Assessment (SGA) and sarcopenia assessment had significantly predict the nutritional status of the study patients (1). Our study results, however, demonstrated a non-significant correlation between the patients’ NRS and clinical outcomes in the PN group. Despite the observation that nutritional status risk did not seem to influence PN decision-making, a similar distribution of at-risk and high-risk patients between the PN and non-PN groups were found, it is important to consider that the decision to initiate PN is multifaceted and may be influenced by additional clinical factors beyond nutritional risk alone.

In addition, the risk of malnutrition in the non-PN group was positively linked with the ICU LOS (*p* = 0.008). Moreover, patients who received PN had significantly longer post-operative, hospital, and ICU LOS (*p* = 0.001) than those who did not receive PN. The ICU admission was also relatively higher in the non-PN group than the PN group. Nutritional status of the patients can also have a significant impact on the outcomes of the surgeries (15, 16). Previous investigations have demonstrated that a preoperative albumin level below 3.5 g/dL has a direct impact on severe morbidity (17). In this study there was a strong significant difference in the albumin level (*p* = 0.029) between the PN and non-PN groups, which could have an influence on the management of the complications for the non-PN group.

The Prognostic Nutritional Index, Nutrition Risk Screening 2002, and Prealbumin represent viable and reliable screening assessments for accurately predicting postoperative nutritional status (18). Malnutrition is a silent and hidden disease that can lead to severe complications for patients who undergo major surgeries (19). Gupta et al. findings have demonstrated that cancer patients often suffer from a decline in nutrition status as a consequence of disease progression and chemotherapy side effects (18). Williams and Wischmeyer have highlighted that cancer patients after major surgeries are at risk of malnutrition (20). Furthermore, Solanki et al. have reported the PN influences the outcomes in terms of length of stay and survival (21). The findings of these studies were consistent with our own findings which demonstrated the relationship between LOS and PN. This study also revealed a relationship between increased risk of malnutrition and ICU LOS, demonstrating a significant correlation between the patients’ NRS and clinical outcomes (*p* = 0.008). Moreover, Kim et al. have suggested the association between preoperative malnutrition status and poor outcomes. The researchers demonstrated that the recovery group who were receiving good nutrition pre- and post-operatively demonstrated an increase in their nutrition status in a period of 3 to12 months after the operation, while the patients who were suffering from malnutrition pre- and post-operation demonstrated a decreased status of nutrition during the same period. The studies also suggest that BMI scores postoperatively remained relatively low in all the groups (malnourished, risk-of-malnutrition, and well-nourished groups) after the 12 months period compared with preoperative levels (21.3 ± 2.8 vs. 22.1 ± 2.5 vs. 24.5 ± 2.4, respectively; *p* < 0.001) (22). Dineen et al. (23) reported similar findings. Nevertheless, our study revealed that the BMI scores for the non-PN group were higher than those of the PN group, although the difference did not achieve statistical significance (*p* = 0.326).

Efficient nutrition routine screening prior to any operation plays a key role in enhancing patients’ clinical outcomes (20). Parenteral nutrition provides cancer patients with essential macronutrients and micronutrients, that contribute to nitrogen balance, muscle mass, surgical recovery time, and immunological function. Furthermore, PN helps minimize complications and the incidence of PN- and non-PN-related complications (24). In the present study, patients in the PN group experienced a variety of complications, including hepatic cholestasis and steatosis, with approximately 50% of PN patients developing cholestasis. PN was also associated with hyperglycemia in 35% of the patients and, to a lesser extent, hypertriglyceridemia in 10%. Although non-PN complications occurred in both groups, the frequency of catheter-related bloodstream infection (*p* = 0.006), wound-related infection (*p* = 0.002), bacteremia (*p* = 0.014), leukopenia (*p* = 0.012), intra-peritoneal abscess (*p* = 0.009), acute kidney injury, and azotemia were more prevalent in the PN group. In terms of mortality rates, our study findings indicate that death cases in the PN group were considerably higher than in the non-PN group (*p* = 0.047). Only one prospective study in malnourished gastric and colorectal cancer patients revealed that preoperative PN led to a lower mortality rate (7 days preoperative PN and 7 days postoperative PN), compared to the control group (2.1% vs. 6%, *p* = 0.003) (24).

This study has several limitations that need to be acknowledged. The study’s retrospective nature prohibits making any inferences about the causal relationship between PN and clinical outcomes. The inclusion of patients from a single medical center limits the generalizability of the findings. The limited duration of the follow-up period restricts the findings to the immediate postoperative period and does not provide insight into postoperative nutrition decline. The NRS-2002 is susceptible to bias due to its emphasis on the assessor’s capacity to collect and interpret data. Despite these limitations, this study, to the best of our knowledge, represents a pioneering effort within the local context examining the prognostic significance of nutritional assessment and intervention in cancer patients undergoing CRS and HIPEC. This highlights the necessity for robust, high-quality randomized controlled trials in this field.

In conclusion, routine preoperative nutrition assessment is essential for identifying patients who are at increased nutritional risk, and nutrition support should be provided preoperatively. The study demonstrated a significant relationship between nutritional risk and ICU LOS. In comparison to the non-PN group, the PN group experienced more complications. Further randomized clinical trials in these patient population, should investigate the systematic provision of PN to all malnourished patients in the preoperative period for at least 7–10 days, with PN continued in the postoperative period.

## Data availability statement

The raw data supporting the conclusions of this article will be made available by the authors, without undue reservation.

## Ethics statement

The studies involving humans were approved by Institutional Review Board at King Saud University Medical City. The studies were conducted in accordance with the local legislation and institutional requirements. The ethics committee/institutional review board waived the requirement of written informed consent for participation from the participants or the participants' legal guardians/next of kin because there was no direct patient contact or intervention used, the blood collected was obtained from the patients routinely as usual patients' care.

## Author contributions

EA: Conceptualization, Methodology, Project administration, Supervision, Validation, Writing – original draft, Writing – review & editing. NK: Data curation, Formal analysis, Writing – original draft, Writing – review & editing. SoA: Data curation, Investigation, Writing – review & editing. FA: Writing – review & editing. SuA: Writing – review & editing. TB: Writing – review & editing.
